# Avoidance memory requires CaMKII activity to persist after recall

**DOI:** 10.1186/s13041-021-00877-5

**Published:** 2021-11-14

**Authors:** Andressa Radiske, Maria Carolina Gonzalez, Janine I. Rossato, Gênedy Apolinário, João R. de Oliveira, Lia R. M. Bevilaqua, Martín Cammarota

**Affiliations:** 1 Memory Research Laboratory, Brain Institute, Federal University of Rio Grande do Norte, Natal, Brazil; 2 Edmond and Lily Safra International Institute of Neuroscience, Macaíba, Brazil; 3 Department of Physiology, Federal University of Rio Grande do Norte, Natal, Brazil

**Keywords:** Reconsolidation, PTSD, Fear, Hippocampus, Retrieval

## Abstract

Avoidance memory is destabilized when recalled concurrently with conflicting information, and must undergo a hippocampus-dependent restabilization process called reconsolidation to persist. CaMKII is a serine/threonine protein kinase essential for memory processing; however, its possible involvement in avoidance memory reconsolidation has not yet been studied. Using pharmacological, electrophysiological and optogenetic tools, we found that in adult male Wistar rats hippocampal CaMKII is necessary to reconsolidate avoidance memory, but not to keep it stored while inactive, and that blocking reconsolidation via CaMKII inhibition erases learned avoidance responses.

**Supplementary Information:**

The online version contains supplementary material available at 10.1186/s13041-021-00877-5.

Recent learned experiences are converted into long-term memories through a protein synthesis-dependent stabilization process known as consolidation that occurs only once for each memory item [1]. However, long-term memories can be destabilized when recalled and must be reconsolidated to persist [2]. Consolidation and reconsolidation share some neurochemical properties, but are independent processes that can be dissociated at different levels [3]. Avoidance is a normal defensive behavior maintained by fear of punishment. Step-down inhibitory avoidance (SDIA) is a one-trial, hippocampus-dependent learning task suitable for studying fear-motivated avoidance memory in rats [4–6]. As a result of SDIA learning, animals suppress their innate preference for stepping down from a raised platform to avoid a footshock (For details see Additional file 1). SDIA-memory consolidation requires activation of hippocampal Ca2 + /calmodulin-dependent protein kinase II (CaMKII) [7], a major Ca2 + signaling effector highly enriched at the postsynaptic side of glutamatergic synapses where it regulates AMPAR targeting [8, 9], one of the putative mechanisms controlling bidirectional synaptic plasticity during reconsolidation [10]. However, the possible involvement of hippocampal CaMKII in avoidance memory reconsolidation has not yet been studied. To do this, we implanted 3-month-old male Wistar rats (300–350 g; Additional file 1) with infusion cannulas in dorsal CA1 and handled (HAN group) or pre-exposed them to the SDIA training box and left to freely explore it for 5-min once a day for 5 consecutive days (PEX group). This last procedure induces learning of non-aversive SDIA-related information without affecting the strength or persistence of SDIA-memory, but making it prone to hippocampus-dependent destabilization and reconsolidation upon unreinforced recall [11, 12]. One day later, HAN and PEX rats were trained in SDIA (0.8 mA/2 s footshock), and 24-h afterwards subjected to a 40-s-long unreinforced SDIA-memory reactivation session (RA) able to induce SDIA-memory reconsolidation but not SDIA-memory extinction [11]. Intra-dorsal CA1 infusion of the CaMKII inhibitor myristoylated autocamptide-2 related inhibitor peptide (AIP) 5-min after RA caused dose-dependent amnesia in PEX but not in HAN animals (Fig. 1a). The amnesic effect of AIP (10 nmol/µl) persisted for at least 7 days (Fig. 1b), was time-dependent (Fig. 1c), contingent on memory reactivation (Fig. 1d), and independent of footshock intensity (Fig. 1e). AIP did not cause amnesia when PEX rats were tested for retention 3-h post-RA (Fig. 1f), when they were submitted to a single pre-exposure session instead of five (Fig. 1g), or when RA lasted 5-s instead of 40-s (Fig. 1h). Dorsal-CA1 αCaMKII Thr-286 autophosphorylation, which is usually used as a proxy of CaMKII activity, increased rapidly after RA in PEX, but not in HAN animals (Fig. 1i; full-length blots in Additional file 2: Fig. S1). Hippocampal theta/gamma phase-amplitude coupling (hPAC) is associated with SDIA-memory destabilization in PEX animals, and medial septum inactivation abolishes RA-induced hPAC and impedes memory destabilization [12]. Thus, if hippocampal CaMKII were really required for SDIA-memory reconsolidation, then optogenetic inactivation of the medial septum of PEX rats during RA should prevent the amnestic effect of post-RA AIP administration. To evaluate this hypothesis, SDIA-trained PEX rats expressing yellow light-sensing archaerhodopsin T in the medial septum (Fig. 1j), whose stimulation reduces basal hippocampal theta power without affecting gamma power (Fig. 1k) and blocks the RA-induced increase in theta power ratio and hPAC (Fig. 1l), were submitted to RA. During this session, the animals were not stimulated or stimulated in the septum with yellow (565 nm) or blue-light (470 nm), and 5-min thereafter received intra-CA1 vehicle or AIP. Retention was tested one day later. As shown in (Fig. 1m), AIP caused amnesia in non-stimulated animals as well as in animals stimulated with blue-light, but not in animals stimulated with yellow-light. Per se, yellow-light stimulation did not affect retention and, when applied immediately after RA, had no effect on the amnesic action of AIP (Fig. 1m). Next, we took advantage of the fact that SDIA memory consolidation, but not the additional learning induced by a second SDIA-training session, requires hippocampal protein synthesis [13] to analyze whether the amnesia caused by CaMKII inhibition is due to impaired recall or storage deficit. We found that SDIA-trained PEX animals that received AIP (10 nmol/µl) after RA reacquired the avoidance response when retrained one day later, but post-retraining intra-CA1 infusion of the protein synthesis inhibitor anisomycin (ANI; 160 µg/µl) impeded reacquisition as if the animals had to consolidate the SDIA response again (Fig. 1n–o).

**Fig. 1:**
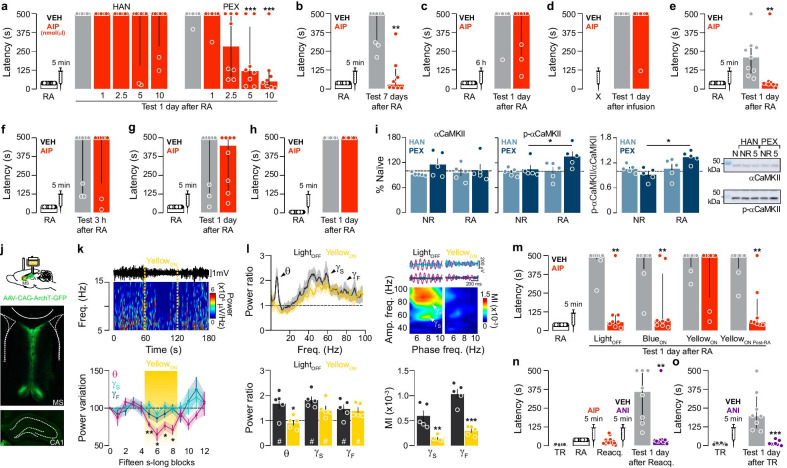
*Hippocampal CaMKII is required for fear-motivated avoidance memory reconsolidation.* **a** HAN and PEX SDIA-trained animals submitted to RA received intra-CA1 VEH or AIP 5-min post-RA and retention was tested 24-h later. **b**–**h** PEX-animals were treated as in **a**, except that **b** memory was evaluated 7-days post-RA; **c** VEH or AIP were given 6-h post-RA; **d** RA was omitted; **e** a 0.4-mA/2-s footshock was given at TR; **f** memory was evaluated 3-h post-RA; **g** they were submitted to single SDIA-preexposure session; **h** RA lasted 5-s. **i** HAN and PEX SDIA-trained animals were or were not (NR) submitted to RA 24-h post-training and 5-min later killed by decapitation to determine dorsal-CA1 αCaMKII and p-Thr286 αCaMKII levels by immunoblotting. **j** Microphotographs showing GFP-reported archaerhodopsin-T expression. **k** Representative spectrogram and normalized theta (θ), slow-gamma (γS) and fast-gamma (γF) power showing the effect of yellow-light MS stimulation on dorsal-CA1 LFPs. **l** *Left.* Mean power ratio (1–100 Hz) during RA for PEX-animals expressing archaerhodopsin-T; bold lines: group mean, shaded areas: SEM. *Right.* Filtered LFP, comodulograms and theta/gamma modulation index (MI). **m** SDIA-trained PEX-animals expressing archaerhodopsin-T were submitted to RA during which the MS was not stimulated (Light_OFF_) or stimulated with blue-light (Blue_ON_) or yellow-light (Yellow_ON_). An additional group received 40-s-long yellow-light immediately after RA (Yellow_ON Post-RA_). Following these manipulations, rats received intra-dorsal-CA1 VEH or AIP. Retention was evaluated 24-h later. **n** PEX-animals trained in SDIA (0.4-mA/2 s) received AIP 5-min post-RA. One day later, the animals were subjected to a test session and when they stepped-down from the platform, they were retrained (Reacq.; 0.4-mA/2 s) and received intra-CA1 VEH or ANI 5-min thereafter. Retention was evaluated 24-h later. **o** PEX-animals trained in SDIA (0.4-mA/2 s) received intra-CA1 VEH or ANI 5-min thereafter and retention was evaluated 24-h later. #p < 0.05 in one-sample Student’s t-test, theoretical mean = 1. Data expressed as median ± IQR or mean ± SEM. *p < 0.05, **p < 0.01, ***p < 0.001. Details of the statistical analyses are presented in Additional file 1: Table S1

Our data confirm that unreinforced recall destabilizes SDIA-memory only when this memory was acquired in a known non-aversive environment, show that hippocampal CaMKII is necessary for SDIA-memory reconsolidation, and suggest that preventing this process by inhibiting CaMKII results in memory erasure. These results differ from reports showing that reduced expression of the CaMKII endogenous inhibitor CaMK2N1 impairs reactivated contextual fear conditioning (CFC) memory persistence, which is associated with decreased hippocampal αCaMKII Thr-286 autophosphorylation two hours after reactivation [14], and that post-recall intra-amygdala administration of AIP does not impair CFC-memory but blocks its destabilization [15]. However, it should be noted that although they may appear similar, CFC and SDIA are fundamentally different learning paradigms, and therefore such conflicting findings should not be surprising. Unlike SDIA, CFC reconsolidation-specific mechanisms cannot be easily distinguished from those engaged merely as a consequence of memory recall; moreover, as the CFC reactivation protocol is methodologically identical to an extinction session, the effect of CaMK2N1 knock-down on the persistence of reactivated CFC memory described in [14] cannot be unequivocally attributed to reconsolidation or extinction regulation, as the authors themselves recognize, even less so when it has been suggested that hippocampal CaMKII is necessary for CFC-memory extinction [16]. A previous report indicates that CaMKII is necessary for recall-induced CFC destabilization in the amygdala [15]. However, we could not study the possible involvement of hippocampal CaMKII on SDIA-memory destabilization because pre-recall intra-CA1 AIP administration hinders memory expression (Additional file 3: Fig. S2) and post-reactivation intra-CA1 AIP is amnestic, which prevented us to analyze whether hippocampal CaMKII inhibition impedes the amnesia provoked by protein synthesis inhibitors. Post-traumatic stress disorder (PTSD) is a mental health condition triggered by experiencing or witnessing a terrifying event and characterized by intrusive thoughts and flashbacks of the traumatic experience. Because learning-based PTSD models posit that classical conditioning is central to the onset of this disorder, most of our knowledge on memory reconsolidation and its possible therapeutic implications derive from CFC studies. However, CFC does not rely on decision-based avoidance responses and hence cannot be used to study the exacerbated avoidance of trauma reminders, a key diagnostic symptom of PTSD that is much better modelled by SDIA and other avoidance learning tasks, such as the shuttle-box avoidance and the platform-mediated avoidance paradigms. Our findings are important in this regard, inasmuch as they indicate that reconsolidation of the mnemonic components of a traumatic experience may be differentially susceptible to pharmacological treatments, which should be taken into consideration when planning reconsolidation-interfering psychotherapeutic strategies.

## Supplementary Information

**Additional file 1.** Extended materials and methods, detailed information of statistics, and step-down latencies during SDIA training.

**Additional file 2.** Full-length versions of blots shown in Fig. 1.

**Additional file 3.** Pre-recall intra-CA1 AIP administration impairs SDIA memory recall but not maintenance.

## Data Availability

Please contact the corresponding author for data requests.

## Abbreviations

AIP: CaMKII inhibitor myristoylated autocamptide-2 related inhibitor peptide
AMPAR: α-Amino-3-hydroxy-5-methyl-4-isoxazolepropionic acid receptor
ANI: Anisomycin
CaMKII: Ca2 + /calmodulin-dependent protein kinase II
CFC: Contextual fear conditioning
HAN: Animals handled for 5 min/day for 5 days
hPAC: Hippocampal theta/gamma phase-amplitude coupling
MS: Medial septum
PEX: Animals allowed to freely explore the SDIA training box for 5 min/day for 5 days
PTSD: Post-traumatic stress disorder
RA: Reactivation session
SDIA: Step-down inhibitory avoidance
