# Supplementary material for: Avoidance memory requires CaMKII activity to persist after recall
Source: Mol Brain. 2021 Nov 14;14:167. doi: 10.1186/s13041-021-00877-5 (PMC8591931; doi:10.1186/s13041-021-00877-5)
Supplement: Supplementary file 1 — Additional file 1. Extended materials and methods, detailed information of statistics, and step-down latencies during SDIA training. [file 13041_2021_877_MOESM1_ESM.docx]

**Avoidance memory requires CaMKII activity to persist after recall**

Andressa Radiske^1^, Maria Carolina Gonzalez^1,2^, Janine I. Rossato^1,3^, Gênedy Apolinário^1^, João R. de Oliveira^1^, Lia R. Bevilaqua^1^, and Martín Cammarota^1^*

^1^Memory Research Laboratory - Brain Institute and ^2^Edmond & Lily Safra International Institute of Neuroscience, Brazil, ^3^Department of Physiology, Federal University of Rio Grande do Norte, Brazil, and *Corresponding author at martin.cammarota@neuro.ufrn.br.

**Materials and Methods**

*Subjects:* We utilized 370 3-month-old naïve male Wistar rats weighing 300-350 g, which were housed in groups of 5 and maintained at 23°C with free access to food and water in the institutional vivarium under a 12:12-h light/dark cycle (lights on at 06:00 AM). Experiments were performed during the light phase of the cycle and followed the NIH Guidelines for Animal Care. Procedures were approved by the local ethics committee. *Surgical procedures:* Rats were anesthetized with a mixture of ketamine (80 mg/kg) and xylazine (10 mg/kg) and implanted with 22-gauge stainless steel guide cannulas into the CA1 region of the dorsal hippocampus (AP, −4.2; LL, ±3.0; DV, −3.0 in mm). For optogenetic experiments, the adeno-associated viral vector AAV-CAG-ArchT-GFP (UNC Vector Core; 2 ×10^11^ particles/ml) was delivered at three different depths in the medial septum using an infusion pump (AP, -0.2; LL, -1.1; DV,- 5.5/-6.0/-6.5 in mm; 10° angle insertion). Two weeks later, optical fibers (200-μm diameter) were implanted in the same region. For LFP recordings, animals were implanted with 16-channel electrode arrays (50 μm tungsten wires coated with PFA arranged in a 2x8 250 μm-spaced configuration) in the CA1 region of the dorsal hippocampus (AP, −3.6; LL, +2.4; DV, −3.6 mm). Two screws were twisted into the parietal bone for ground connections. After surgery, animals received subcutaneous meloxicam (0.2 mg/kg). Rats implanted with electrodes were housed individually. Experiments began no less than 10-d after surgery. *Drugs and infusion procedures:* Myristoylated AIP bought from Sigma-Aldrich was dissolved in sterile saline (vehicle; pH ~7.2) upon arrival, aliquoted and stored at -20°C. Stock aliquots were thawed and diluted to working concentration in vehicle on the day of the experiment. The dose of AIP used was based on previous studies and pilot experiments [1-4]. For drug delivery, infusion cannulas were fitted into the guide cannulas and infusions were carried out using a Hamilton syringe coupled to an infusion pump (1 μl/side at 0.5 μl/min). Infusion cannulas were left in place for one additional minute to minimize backflow. Cannula placement was verified postmortem one day after the last behavioral test. Only data from animals with correct implants were analyzed. *Habituation:* For experiments involving optogenetic stimulation and electrophysiological recordings, animals were habituated to mounting the optic fiber and the headstage without anesthesia as well as to move freely in the recording cage with cables and optical fibers attached to the implants. *Step-down inhibitory avoidance (SDIA) task:* The training apparatus consisted of a Plexiglas box (50 x 25 x 25 cm) with an elevated wood platform (5 x 8 x 25 cm) positioned on the left end of the box metal grill floor. Before training, rats were handled 5-min per day for 5 consecutive days (HAN animals) or allowed to freely explore the training box 5-min per day for 5 consecutive days (PEX animals). One day after the end of these procedures, animals were trained in the SDIA task. To do that, they were gently placed on the training box platform and received a 0.8 mA/2-s scrambled footshock when stepped down to the grid. Some animals were trained using a weaker footshock (0.4 mA/2-s). Immediately after training, animals were returned to their home cages and 24-h later placed again on the training box platform for 40-s to reactivate the SDIA memory. During the SDIA memory reactivation session the animals explored the platform avoiding stepping down from it. We did not observe any significant behavioral difference between HAN and PEX animals during the reactivation session. SDIA memory retention was evaluated 1-d after training or reactivation by placing the animals on the training box platform and measuring their latency to step down. The test session finished when the rats stepped down to the grid (footshock omitted) or after 500-s. All animals were trained and tested only once, except when mentioned. *Optogenetic stimulation:* For optogenetic experiments, rats were injected with AAV-CAG-ArchT-GFP and implanted with an optic fiber into the medial septum, as described above. Optic fibers were coupled to blue-light (470 nm) or yellow-light (565 nm) emitting LEDs (ThorLabs). Light was applied using a DC4104 LED driver (ThorLabs). *In vivo electrophysiology:* The Cerebus Neural Signal processor system (BlackRock Microsystems) was used to record extracellular signals. Electrophysiological data were acquired at 1kHz, filtered (0.3-250 Hz), amplified, digitized, and stored for offline analyses. Data were analyzed in Matlab (MathWorks, USA) using built-in and custom-written routines from the Signal Processing Toolbox. Baseline signal recordings were acquired in a recording cage for 30 min before memory reactivation. We used the first 40-s of stable signals during which the animal was awake and in a minimal movement state. Power spectra were calculated using the Welch's periodogram (4-s Hamming windows, 75% overlap). Spectrograms were calculated using sliding windows of 4-s and 1-s-long steps. Theta (θ; 5-10), slow gamma (γS; 35-55) and fast gamma (γF; 65-100) band powers were defined as the average power in the frequency range. To analyze the effect of yellow light stimulation on spontaneous hippocampal oscillatory activity, power variation was calculated as percent change from the baseline. Theta/gamma phase–amplitude cross-frequency coupling was computed from the Hilbert transform of the filtered versions of theta phase and gamma amplitude frequency bands. Theta phase was binned into 18 intervals of 20° and the mean amplitude of gamma bands was computed for each theta phase bin and normalized by the sum of amplitude values over all bins. The modulation index (MI) expresses the strength of theta/gamma phase–amplitude cross-frequency coupling. Comodulation maps were obtained by expressing the MI of several frequency band pairs (4 Hz bandwidths, 1 Hz steps for phase frequencies; 10 Hz bandwidths, 2 Hz steps for amplitude frequencies) in a bidimensional pseudocolor plot. Mean MIs were defined by averaging the corresponding MI values in the (5–10 Hz) × (35–55 Hz) or (5–10 Hz) × (65–100 Hz) regions of the comodulation maps. To calculate MI, we used LFP recordings from the entire 40-s-long reactivation session. Video cameras fixed above the SDIA apparatus were used to record the animal behavior. Video data were acquired at 30 frames/s and analyzed using the TopScan system. During the reactivation session, all animals stayed on the platform (mean velocity <1 cm/s), thus excluding the possibility that speed-dependent variations in hippocampal LFP activity account for our results. *Immunoblotting:* The CA1 region of the dorsal hippocampus was dissected out and homogenized in ice-chilled homogenization buffer (20 mM Tris-HCl, pH 7.4, containing 0.32 M sucrose, 1 mM EDTA, 1 mM EGTA, 1 mM PMSF, 10 μg/ml aprotinin, 15 μg/ml leupeptin, 10 μg/ml bacitracin, 10 μg/ml pepstatin, 50 mM NaF, and 1 mM sodium orthovanadate). Protein concentration was estimated using the BCA protein assay (Pierce) and equal amounts of proteins fractionated by SDS-PAGE before being transferred to PVDF membranes (Immobilon-P, Millipore). After verification of protein loading by Ponceau S staining, the blots were blocked in Tween Tris-HCl buffer saline (TTBS; 100 mM Tris-HCl, pH 7.5, containing 0.9% NaCl and 0.1% Tween 20) and incubated overnight with anti-pThr286 αCaMKII (1:20000 dilution, Cell Signaling Technology, Inc.). The blots were washed in TTBS and incubated with HRP-coupled anti-IgG antibodies, washed again, and immunoreactivity detected using the ECL Prime Western Blotting Detection Reagent (GE Healthcare Amersham). Densitometric analyses were performed with an ImageQuant RT-ECL system (GE Healthcare). After that the PVDF membranes were stripped and blotted with anti-αCaMKII (1:10000 dilution, Santa Cruz Biotechnology). *Immunofluorescence:* Rats were transcardially perfused with 4% PFA, at pH ∼7.2. Brains were removed, placed in PFA at 4°C overnight, transferred to 30% sucrose, and allowed to settle for 48-h. Brains were cut in 50-µm coronal sections in a cryostat. Free-floating slices from the rostral-caudal extent of the dorsal hippocampus and the medial septum were rinsed with PBS, incubated in PBST (0.2% Triton X-100) for 1 h, and blocked for 2 h at room temperature. Slices were incubated with anti-GFP (1:1000 dilution, Abcam) overnight at 4°C and then washed in PBST and incubated with Alexa Fluor 488 anti-chicken (1:1000 dilution, Thermo Fisher Scientific) for 2-h at room temperature. Slices were counterstained with DAPI (1:1000 dilution, Thermo Fisher Scientific), mounted with Fluoromount-G, and stored at −20°C until image acquisition. Images were acquired using a Leica DM5000 fluorescence microscope and a CoolSNAP HQ2 CCD Camera in 16-bit grayscale. For visualization, pseudocolor was applied (linear look-up table covered full images, Image-Pro Plus 7.0 software). *Statistical analysis:* Data were analyzed using GraphPad Prism 8 and presented as mean ± standard error of mean or median ± interquartile range. Significance was set at p < 0.05. The number of subjects per group / sample size was based on previous reports and is indicated in the figures (dots). Subjects were randomly assigned to experimental groups. Step-down latencies during SDIA training were expressed as mean ± SEM and analyzed using unpaired Student's t test (Table 2). Because of the 500-s ceiling imposed, step-down latencies were analyzed by two-tailed Mann–Whitney U test. Immunoblots and electrophysiological data were analyzed using a one-sample t test with a theoretical mean of 1, paired t test, unpaired Student's t test, one-way repeated-measures (RM) ANOVA, or two-way ANOVA followed by Bonferroni's multiple-comparisons test.

| **Table 1. Statistical analyses** | | | | | |
| --- | --- | --- | --- | --- | --- |
| **Figure** | **Statistical method** | **n** | **Statistic details** | | **Post-hoc multiple comparisons test** |
| 1 a | Two-way ANOVA | HAN  n = 8 per treatment;  PEX  n = 8 per treatment | Dose effect:  F(4, 35) = 10.42 | p < 0.0001 | Bonferroni  PEX VEH vs PEX AIP 5 or 10  p < 0.001 |
|  |  |  | Pre-exposition effect:  F(1, 35) = 23.80 | p < 0.0001 |  |
|  |  |  | Interaction:  F(4, 35) = 3.470 | p = 0.0173 |  |
| 1 b | Mann–Whitney test | VEH n = 8  AIP n = 8 | U = 3 | p = 0.0011 | - |
| 1 c | Mann–Whitney test | VEH n = 8  AIP n = 8 | U = 24.5 | p = 0.4462 | - |
| 1 d | Mann–Whitney test | VEH n = 8  AIP n = 8 | U = 28 | p > 0.9999 | - |
| 1 e | Mann–Whitney test | VEH n = 9  AIP n = 9 | U = 8.5 | p = 0.0028 | - |
| 1 f | Mann–Whitney test | VEH n = 8  AIP n = 8 | U = 31 | p > 0.9999 | - |
| 1 g | Mann–Whitney test | VEH n = 8  AIP n = 8 | U = 31 | p = 0.9608 | - |
| 1 h | Mann–Whitney test | VEH n = 9  AIP n = 8 | U = 36 | p > 0.9999 | - |
| 1 i | Two-way ANOVA | αCaMKII  HAN  n = 5 NR  n = 5 RA  PEX  n = 5 NR  n = 5 RA | Reactivation effect:  F (1, 8) = 0.5415 | p = 0.4828 | - |
|  |  |  | Pre-exposition effect:  F (1, 8) = 1.370 | p = 0.2755 |  |
|  |  |  | Interaction:  F (1, 8) = 1.758 | p = 0.2215 |  |
|  | Two-way ANOVA | pThr-286 αCaMKII  HAN  n = 5 NR  n = 5 RA  PEX  n = 5 NR  n = 5 RA | Reactivation effect:  F (1, 8) = 8.124 | p = 0.0215 | Bonferroni  PEX RA vs PEX NR  p < 0.05 |
|  |  |  | Pre-exposition effect:  F (1, 8) = 3.489 | p = 0.0987 |  |
|  |  |  | Interaction:  F (1, 8) = 5.733 | p = 0.0436 |  |
|  | Two-way ANOVA | pThr-286 αCaMKII/αCaMKII HAN  n = 5 NR  n = 5 RA  PEX  n = 5 NR  n = 5 RA | Reactivation effect:  F (1, 8) = 7.793 | p = 0.0235 | Bonferroni  PEX RA vs PEX NR  p < 0.05 |
|  |  |  | Pre-exposition effect:  F (1, 8) = 0.9334 | p = 0.3623 |  |
|  |  |  | Interaction:  F (1, 8) = 7.358 | p = 0.0266 |  |
| 1k | RM one-way ANOVA | Theta power  variation  n = 5 | F (2.869, 17.22) = 3.348 | p = 0.0451 | Bonferroni  Baseline vs 5  p < 0.01;  Baseline vs 6, 7, 8  p < 0.05 |
|  |  | Slow gamma variation  n = 5 | F (1.675, 10.05) = 0.6269 | p = 0.5268 | - |
|  |  | Fast gamma variation  n = 5 | F (2.945, 17.67) = 1.582 | p = 0.2295 | - |
| 1 l *Left* | Unpaired t test | Theta power Light_OFF_ n = 5  Light_ON_ n = 5 | t(8) = 3.145 | p = 0.0137 | - |
|  |  | Slow gamma power Light_OFF_ n = 5  Light_ON_ n = 5 | t(8) = 1.827 | p = 0.1050 | - |
|  |  | Fast gamma power  Light_OFF_ n = 5  Light_ON_ n = 5 | t(8) = 0.3230 | p = 0.7550 | - |
| 1 l *Right* | Unpaired t test | Theta/Slow gamma  MI  Light_OFF_ n = 5 Light_ON_ n = 5 | t(8) = 4.166 | p = 0.0031 | - |
|  |  | Theta/Fast gamma  MI  Light_OFF_ n = 5; Light_ON_ n = 5 | t(8) = 7.486 | p < 0.0001 | - |
| 1 m | Mann–Whitney test | Light_OFF_  VEH n = 8  AIP n = 8 | U = 5 | p = 0.0020 | - |
|  |  | Blue_ON_  VEH n = 8  AIP n = 8 | U = 7 | p = 0.0056 | - |
|  |  | Yellow_ON_  VEH n = 8  AIP n = 8 | U = 30 | p = 0.7128 | - |
|  |  | Yellow_ON Post-RA_  VEH n = 8  AIP n = 8 | U = 6 | p = 0.0033 | - |
| 1 n | Mann–Whitney test | VEH n = 8  ANI n = 9 | U = 6.50 | p = 0.0025 | - |
| 1 o | Mann–Whitney test | VEH n = 8  ANI n = 8 | U = 0 | p = 0.0002 | - |

**Table 1. Detailed information of statistics.**

| **Table 2. Step-down latency during SDIA training** | | | | | | |
| --- | --- | --- | --- | --- | --- | --- |
| **Figure** | **Behavioral condition** | **Treatment** | **Latency (s) (per Group)** | **n** | **Latency (s) (per Condition)** | **P (HAN vs PEX)** |
| 1 a | HAN | VEH | 16.63 ± 0.99 | 8 | 16.73 ± 0.86 | < 0.0001 |
|  |  | AIP 1 nmol/ul | 20.13±1.84 | 8 |  |  |
|  |  | AIP 2.5 nmol/ul | 15.25±2.40 | 8 |  |  |
|  |  | AIP 5 nmol/ul | 15.25±2.18 | 8 |  |  |
|  |  | AIP 10 nmol/ul | 16.38±1.76 | 8 |  |  |
|  | PEX | VEH | 5.75±1.03 | 8 | 7.00±0.53 |  |
|  |  | AIP 1 nmol/ul | 6.25±0.88 | 8 |  |  |
|  |  | AIP 2.5 nmol/ul | 6.62±1.63 | 8 |  |  |
|  |  | AIP 5 nmol/ul | 7.37±0.46 | 8 |  |  |
|  |  | AIP 10 nmol/ul | 9.00±1.48 | 8 |  |  |
| 1 b | PEX | VEH | 7.12±0.74 | 8 |  |  |
|  |  | AIP | 5.50±0.68 | 8 |  |  |
| 1 c | PEX | VEH | 6.62±1.47 | 8 |  |  |
|  |  | AIP | 6.70±0.86 | 8 |  |  |
| 1 d | PEX | VEH | 8.37±0.65 | 8 |  |  |
|  |  | AIP | 7.75±1.66 | 8 |  |  |
| 1 e | PEX | VEH | 6.77±1.31 | 9 |  |  |
|  |  | AIP | 7.11±0.84 | 9 |  |  |
| 1 f | PEX | VEH | 6.37±1.13 | 8 |  |  |
|  |  | AIP | 8.87±1.00 | 8 |  |  |
| 1 g | PEX | VEH | 7.12±1.00 | 8 |  |  |
|  |  | AIP | 9.00±1.29 | 8 |  |  |
| 1 h | PEX | VEH | 6.77±1.31 | 9 |  |  |
|  |  | AIP | 7.00±0.94 | 8 |  |  |
| 1 i | HAN | NR | 12.80±1.65 | 5 | 13.10±1.21 | < 0.0001 |
|  |  | RA | 13.40±1.96 | 5 |  |  |
|  | PEX | NR | 6.00±1.04 | 5 | 5.70±0.55 |  |
|  |  | RA | 5.40±0.50 | 5 |  |  |
| 1 l | PEX | LightOFF | 7.00±0.89 | 5 |  |  |
|  |  | LightON | 6.20±0.96 | 5 |  |  |
| 1 m | LightOFF | VEH | 7.00±0.88 | 8 |  |  |
|  |  | AIP | 7.75±1.22 | 8 |  |  |
|  | BlueON | VEH | 5.50±0.90 | 8 |  |  |
|  |  | AIP | 6.87±0.61 | 8 |  |  |
|  | YellowON | VEH | 7.37±0.92 | 8 |  |  |
|  |  | AIP | 5.75±0.88 | 8 |  |  |
|  | YellowON-Post RA | VEH | 7.50±1.11 | 8 |  |  |
|  |  | AIP | 7.37±1.06 | 8 |  |  |
| 1 n | PEX | VEH | 8.00±0.70 | 8 |  |  |
|  |  | ANI | 7.22±0.59 | 9 |  |  |
| 1 o | HAN | VEH | 16.75±1.60 | 8 |  |  |
|  |  | ANI | 15.63±1.59 | 8 |  |  |
| S2 | PEX | VEH | 4.11±0.35 | 9 |  |  |
|  |  | AIP | 5.50±0.53 | 8 |  |  |

**Table 2. Step-down latency during SDIA training.** Pre-exposure to the SDIA training box decreased step-down latency at training, indicating learning of SDIA-related nonaversive information during pre-expositions [5]. Latencies did not differ between groups for the same behavioral condition. Data are presented as the mean ± SEM and were analyzed using an unpaired t test.

**Reference**

1. Gonzalez MC, Rossato JI, Radiske A, Bevilaqua LRM, Cammarota M. Dopamine controls whether new declarative information updates reactivated memories through reconsolidation. Proc Natl Acad Sci U S A. 2021;118(29):e2025275118.

2. Bian H, Yu LC. Intra-nucleus accumbens administration of the calcium/calmodulin-dependent protein kinase II inhibitor AIP induced antinociception in rats with mononeuropathy. Neurosci Lett. 2015;599:129-32.

3. Gómez-Pinilla F, Huie JR, Ying Z, Ferguson AR, Crown ED, Baumbauer KM, Edgerton VR, Grau JW. BDNF and learning: Evidence that instrumental training promotes learning within the spinal cord by up-regulating BDNF expression. Neuroscience. 2007;148(4):893-906.

4. Liu Z, Zhang JJ, Liu XD, Yu LC. Inhibition of CaMKII activity in the nucleus accumbens shell blocks the reinstatement of morphine-seeking behavior in rats. Neurosci Lett. 2012;518(2):167-71.

5. Radiske A, Gonzalez MC, Conde-Ocazionez SA, Feitosa A, Köhler CA, Bevilaqua LR, Cammarota M. Prior Learning of Relevant Nonaversive Information Is a Boundary Condition for Avoidance Memory Reconsolidation in the Rat Hippocampus. J Neurosci. 2017;37(40):9675-9685.
