# Supplementary figures and images for: Avoidance memory requires CaMKII activity to persist after recall
Source: Mol Brain. 2021 Nov 14;14:167. doi: 10.1186/s13041-021-00877-5 (PMC8591931; doi:10.1186/s13041-021-00877-5)

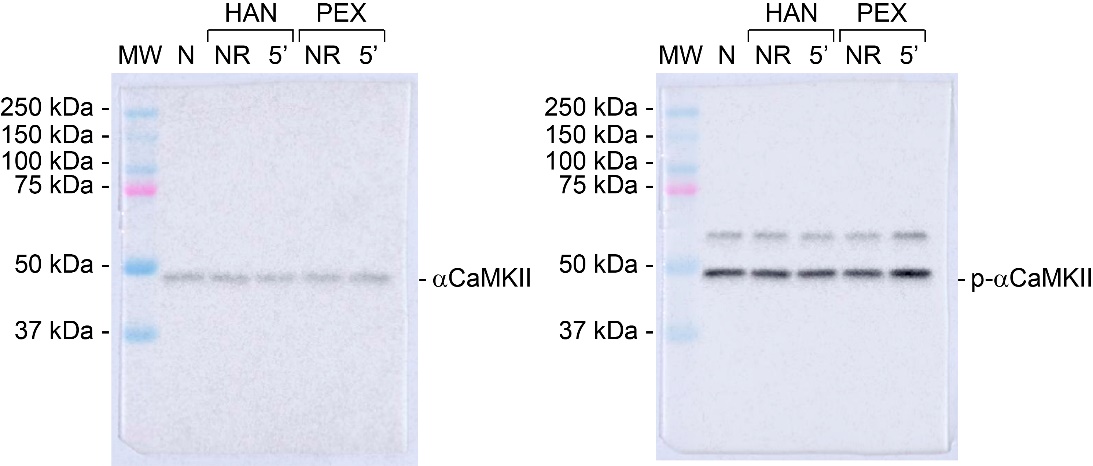


**Figure S1. Full-length versions of the blots shown in Fig 1.**

Supplement: Supplementary file 2 — Additional file 2. Full-length versions of blots shown in Fig. 1. [file 13041_2021_877_MOESM2_ESM.docx]
