# Supplementary material for: Avoidance memory requires CaMKII activity to persist after recall
Source: Mol Brain. 2021 Nov 14;14:167. doi: 10.1186/s13041-021-00877-5 (PMC8591931; doi:10.1186/s13041-021-00877-5)
Supplement: Supplementary file 3 — Additional file 3. Pre-recall intra-CA1 AIP administration impairs SDIA memory recall but not maintenance. [file 13041_2021_877_MOESM3_ESM.docx]

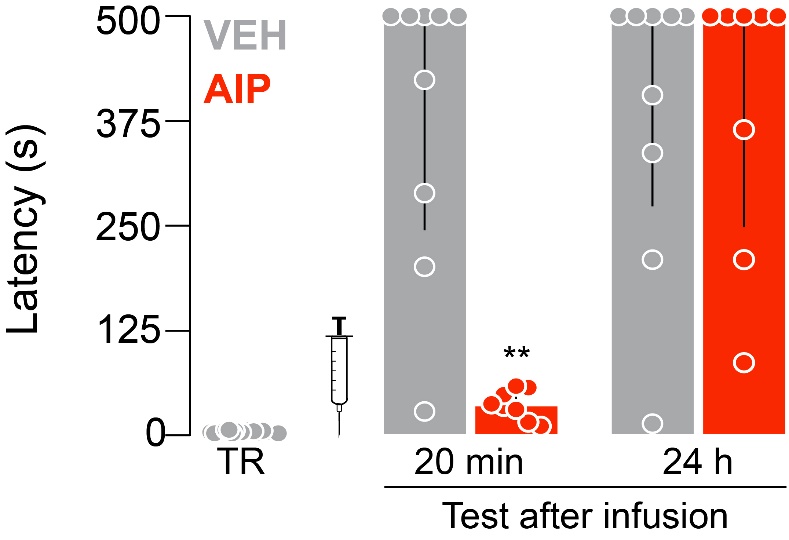


**Figure S2. Pre-recall intra-CA1 AIP administration impairs SDIA memory recall but not maintenance.** PEX rats were trained (TR; 0.8 mA/2 s) in SDIA 24-h after the last pre-exposition session and 24-h later received bilateral intra-CA1 infusions of VEH or AIP. Retention was tested twice, 20-min and 24-h post-infusion. 20 min: U=6, **p<0.01, VEH vs AIP in Mann–Whitney test. Data expressed as median ± IQR.
